# Joint associations of serum uric acid and ALT with NAFLD in elderly men and women: a Chinese cross-sectional study

**DOI:** 10.1186/s12967-018-1657-6

**Published:** 2018-10-17

**Authors:** Huanhuan Yang, Deming Li, Xiaochao Song, Fang Liu, Xinjing Wang, Qinghua Ma, Xi Zhang, Xinli Li

**Affiliations:** 1 School of Public Health, Medical College of Soochow University, Suzhou, 215123 Jiangsu China; 2 The Third Hospital of Xiangcheng, Suzhou, Jiangsu China; 3 Clinical Research Unit, Xin Hua Hospital, Shanghai Jiao Tong University School of Medicine, Shanghai, 200092 China; 4 Jiangsu Key Laboratory of Preventive and Translational Medicine for Geriatric Diseases, School of Public Health, Soochow University, Suzhou, China

**Keywords:** Alanine aminotransferase, Gender difference, Non-alcoholic fatty liver disease, Uric acid

## Abstract

**Background:**

Epidemiological evidence suggests sex difference in serum uric acid (SUA) and alanine aminotransferase (ALT) might be a potential explanation for the gender difference in prevalence of non-alcoholic fatty liver disease (NAFLD). However, few epidemiology data in China have tested this hypothesis.

**Methods:**

We conducted a cross-sectional study to assess the joint associations between SUA and serum ALT with NAFLD among elderly Chinese men and women.

**Results:**

Among 7569 participants with a mean age of 59.8 years (± 13.4 years), 56.6% of women and 43.4% of men were diagnosed as NAFLD, respectively. A positive association between SUA and NAFLD prevalence was observed in both men and women. NAFLD prevalence was 2.74 times (95% CI 2.00–3.76) higher for men and 4.60 times (95% CI 3.39–6.24) higher for women with the highest quintiles of SUA levels compared to those with the lowest levels. SUA levels were significantly associated with prevalence of mild- and severe-steatosis (*P *< 0.01). In addition, the ORs of NAFLD among participants with high SUA levels and high serum ALT was 10.75 (95% CI 3.56–32.46) for men and 7.96 (95% CI 2.83–22.39) for women, compared with those with low SUA levels and low serum ALT.

**Conclusions:**

SUA levels were positively associated with NAFLD prevalence, and the association was slightly stronger in women than in men. A significant joint association of SUA and serum ALT with NAFLD prevalence was observed in all participants, which was slightly stronger in men than in women.

## Background

Non-alcoholic fatty liver disease (NAFLD), a multi-factorial disease, was formed by the interactions of genetics, diet and lifestyle [1]. Increasing observational evidence suggests a close relationship between serum uric acid (SUA) levels and risk of NAFLD [2–4]. Uric acid is the major end product of purine metabolism, and the imbalance of uric acid formation and excretion might result in high levels of SUA in human body [5]. Several human studies found a high activity of serum xanthine oxidoreductase in NAFLD patients, which catalyzes formation of uric acid, and then the increased generation of uric acid is able to accelerate the development of NAFLD mediated by xanthine oxidoreductase [6]. Elevated uric acid levels could induce the triglyceride accumulation by promoting the over-expression of pro-lipogenic enzymes sterol regulatory element-binding proteins [7]. Moreover, uric acid usually conserves the strong oxidant activity in patients with metabolic syndrome [8]. Meanwhile, NAFLD is generally regarded as a hepatic manifestation of the metabolic syndrome, thus, the pro-oxidative effects of uric acid could promote the progression of liver injury in NAFLD patients [9]. Additionally, there is an indirect effect of elevated SUA levels on NAFLD risk by inducing insulin resistance [10–13]. An animal study demonstrated that anti-uric acid treatment for 18 weeks in obese mice could completely reverse the fatty liver condition [14].

Alanine aminotransferase (ALT), a hepatic enzyme, is mostly presented in the liver and commonly used for indicating the presence of liver disease. ALT is a specific marker of liver inflammation and hepatocellular injury [15]. Recent observational studies suggest that liver damage already exist even when the level of ALT is within the normal range. Thus, a diagnosis by elevated levels of serum ALT might underestimate the prevalence of NAFLD [16–18]. Evidence from epidemiological studies indicates a sex difference in NAFLD prevalence in the elderly. A lower prevalence was observed in women than in men, 31.77% for men and 20.59% for women [19]. The prevalence rises from 22.07% after the age of 50 years, reaches to the peak of 30.79% at 60–69 years, and declines after 70 years for women [19], while the prevalence of NAFLD for men reaches to the peak of 27.69% at 40–49 years, then declines after the age of 50 years with an inverted U-shaped relation [19, 20]. A population-based cross-sectional study among 82,608 adults aged 43.91 (± 10.15) years in Israel found a stronger association between uric acid and elevated ALT among women than men [21]. The gender difference for the ALT-SUA association possibly contributes to the gender difference for the NAFLD prevalence, and might help to explain the reason why ALT underestimated the NAFLD prevalence.

However, only few studies specifically have evaluated the joint associations of serum SUA levels and ALT with risk of NAFLD in China. Thus, we conducted a population-based cross-sectional study, including 7569 elderly men and women, to evaluate the joint associations of SUA levels and serum ALT on NAFLD prevalence.

## Methods

### Participants

The study was compounded by patients who underwent physical examination at the Third hospital of Xiangcheng during March to December 2015. Participants were excluded if they met the following criteria: (1) taking anti-hypertensive agents, or anti-diabetic, lipid-lowering or hypouricemic agents within 24-h before physical examination; (2) with alcohol consumption greater than 140 g/week for men and 70 g/week for women; (3) with chronic liver disease, such as cirrhosis, liver cancer, viral hepatitis, autoimmune hepatitis, and taking hepatotoxic medicines. A total of 7569 participants, 3126 men and 4443 women, with a mean age of 60 years were finally included in the analyses.

### Clinical examinations and biochemical assays

Clinical examinations included physical examination, measurement of blood pressure (BP), and anthropometry assays. General medical history was collected by face-to-face interview by trained physicians.

Venous blood samples were drawn in the morning after an overnight fasting, and serum samples were separated for the analysis of biochemical assays without frozen. Biochemical variables, including serum ALT, aspartate aminotransferase (AST), triglyceride (TG), total cholesterol (TC), high-density lipoprotein cholesterol (HDL-C), low-density lipoprotein cholesterol (LDL-C), SUA, and fasting plasma glucose (FPG), were analyzed in the Lab of the Third Hospital of Xiangcheng using an Olympus AU640 auto analyzer (Olympus, Kobe, Japan). Standard laboratory assay methods and quality control methods were conducted.

### Diagnosis of NAFLD

NAFLD was diagnosed by abdominal ultrasound according to the conventional criterion [22]. Hepatic ultrasonic examination was performed by trained ultrasonographists who were blinded to the clinical and laboratory data. Hepatic steatosis was diagnosed by characteristic echo patterns according to conventional criteria, such as the evidence of diffuse hyper-echogenicity of the liver relative to the kidneys, ultrasound beam attenuation, and poor visualization of intrahepatic structures [23]. According to the above criteria, steatosis status of NAFLD was further classified into mild-steatosis or severe-steatosis by experienced and trained physicians. Besides the fatty liver, liver cyst and calcification focus were also examined using ultrasound.

### Statistical analyses

We calculated means ± standard deviations (SDs) for continuous variables and frequency (percentage) for categorical variables. The independent-samples *t* tests were performed to compare the differences of continuous variables between men and women, and the categorical variables were compared by using Chi square tests.

We stratified the total population by the quintiles of SUA levels: 230 μmol/L, 271 μmol/L, 312 μmol/L, 366 μmol/L, and 366 μmol/L. The quintiles of SUA levels for men was 278 μmol/L, 317 μmol/L, 355 μmol/L, 402 μmol/L and 403 μmol/L, and for women they were 213 μmol/L, 244 μmol/L, 276 μmol/L, 320 μmol/L and 321 μmol/L. The quartiles of ALT levels were 14 U/L, 18 U/L, 24 U/L and 25 U/L; and for men they were 16 U/L, 19 U/L, 27 U/L and 28 U/L, and for women: 14 U/L, 16 U/L, 23 U/L and 24 U/L. Hyperuricemia was defined as SUA level higher than 420 μmol/L in men and higher than 360 μmol/L in women [24]. Multivariate logistic regression models adjusted for age and sex (Model 1) or age, sex, body mass index (BMI), history of cardiovascular related diseases, diastolic blood pressure (DBP), serum AST, TG, TC, HDL and FPG (Model 2) were used to estimate the odds ratios (ORs) of the associations of ALT and SUA with NAFLD. Cardiovascular related diseases including hypertension, diabetes, coronary heart disease and stroke in current study. To further investigate the joint associations of ALT and SUA with NAFLD, we divided the participants into 4 group according to ALT levels (cutoff point: 40 U/L) and SUA levels (cutoff point: 420 μmol/L for men, 360 μmol/L for women), and binary logistic regression was used to calculate the ORs of this new variable for NAFLD prevalence.

All statistical analyses were conducted using the SPSS software package version 21.0 (SPSS Inc., Chicago, IL). Two-tailed *P* value < 0.05 was considered as statistically significant.

## Results

### Characteristics of participants

**Fig. 1:**
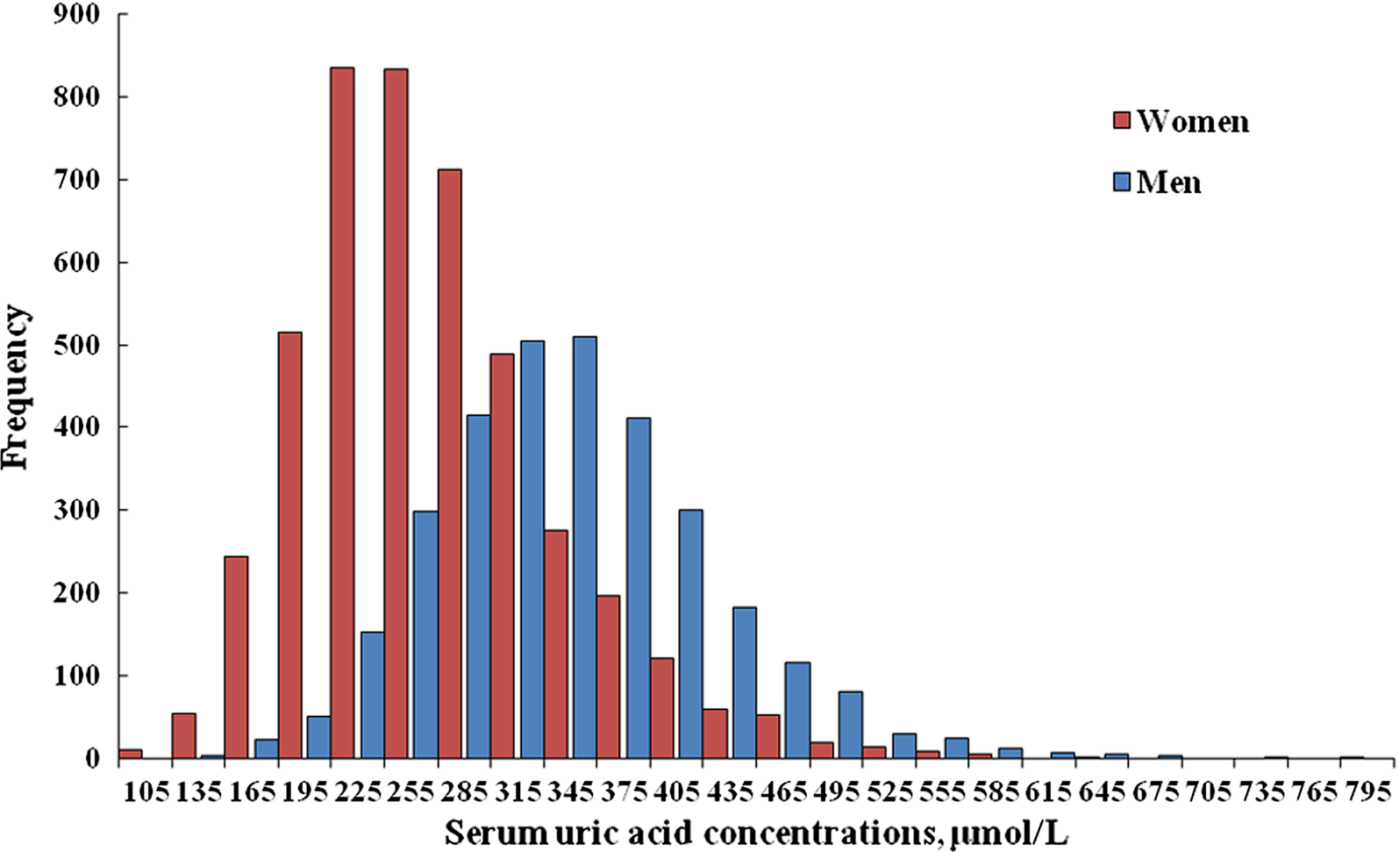
Serum uric acid levels in Chinese men and women

A total of 7569 participants (men: 41.3%) aged 60 (± 13) years with a mean BMI of 23.93 kg/m^2^ (SD: 2.73) were included, the average SUA levels were significantly higher in men than in women, 343 μmol/L (± 77.6) for men and 270 μmol/L (± 70.9) for women (*P*-value < 0.001) (Fig. 1).

Compared to women, men were younger, had lower levels of TC, HDL-C, LDL-C, higher levels of serum AST, ALT, FPG (all *P*-values < 0.01), and especially significantly higher SUA levels (men *vs.* women: 343 ± 77.6 *vs.* 270 ± 70.9 μmol/L, *P*-value < 0.001). The prevalence of NAFLD was 30.93% among men and 28.40% for women (*P*-value = 0.02). 14.7% of men had hyperuricemia and 8.2% were also with hyper-ALT levels (serum ALT levels > 40 U/L) (Table 1).

**Table 1:** Characteristics of participants according to the quintiles of SUA

Variables	Total population			SUA quintiles (For men)					*P*
	Men (n = 3126)	Women (n = 4443)	*P*	Q1 (n = 603)	Q2 (n = 646)	Q3 (n = 617)	Q4 (n = 629)	Q5 (n = 631)	
Age (years)	59.35 ± 9.38	60.19 ± 9.82	< 0.001	59.29 ± 8.68	58.62 ± 9.38	58.42 ± 8.73	59.96 ± 9.91	60.47 ± 9.96	< 0.001
BMI (kg/m2)	23.86 ± 2.96	23.98 ± 3.21	0.090	22.80 ± 2.73	23.50 ± 2.84	24.00 ± 2.69	24.11 ± 3.03	24.85 ± 3.11	< 0.001
SBP (mmHg)	137.23 ± 19.28	141.75 ± 19.93	< 0.001	133.88 ± 19.05	136.15 ± 18.69	137.29 ± 18.98	138.72 ± 19.54	140.00 ± 19.64	< 0.001
DBP (mmHg)	86.56 ± 11.78	84.25 ± 11.66	< 0.001	83.91 ± 11.60	85.84 ± 11.58	86.86 ± 12.23	88.03 ± 12.10	88.10 ± 10.88	< 0.001
TG (mmol/L)	1.32 ± 1.11	1.32 ± 0.81	0.801	1.02 ± 0.66	1.13 ± 0.75	1.35 ± 1.06	1.35 ± 1.03	1.74 ± 1.63	< 0.001
TC (mmol/L)	4.74 ± 0.91	4.91 ± 0.90	< 0.001	4.62 ± 0.82	4.62 ± 0.82	4.78 ± 0.88	4.74 ± 0.90	4.92 ± 1.05	< 0.001
HDL-C (mmol/L)	1.47 ± 0.43	1.51 ± 0.37	< 0.001	1.57 ± 0.42	1.49 ± 0.40	1.45 ± 0.44	1.44 ± 0.46	1.38 ± 0.41	< 0.001
LDL-C (mmol/L)	2.61 ± 0.69	2.75 ± 0.70	< 0.001	2.51 ± 0.63	2.54 ± 0.65	2.66 ± 0.68	2.61 ± 0.70	2.72 ± 0.76	< 0.001
AST (U/L)	24.52 ± 10.99	23.09 ± 9.50	< 0.001	22.07 ± 7.16	23.62 ± 9.53	24.94 ± 10.75	24.84 ± 9.97	27.04 ± 15.23	< 0.001
ALT (U/L)	23.02 ± 15.68	19.99 ± 14.35	< 0.001	18.77 ± 9.76	21.24 ± 12.05	24.03 ± 16.14	24.39 ± 17.53	26.55 ± 19.58	< 0.001
FPG (mmol/L)	6.07 ± 1.32	6.01 ± 1.21	0.036	6.19 ± 1.76	6.04 ± 1.34	6.05 ± 1.24	6.01 ± 1.13	6.06 ± 1.01	0.140
SUA (μmol/L)	343.37 ± 77.58	270.46 ± 70.93	< 0.001	243.31 ± 27.53	298.70 ± 11.49	336.34 ± 10.45	376.99 ± 13.37	458.10 ± 52.17	< 0.001
Hyper-ALT, n (%)	256 (8.2)	240 (5.4)	< 0.001	23 (3.8)	33 (5.1)	64 (10.4)	58 (9.2)	78 (12.4)	< 0.001
NAFLD, n (%)	967 (30.93)	1262 (28.40)	0.017	98 (16.3)	147 (22.8)	197 (31.9)	221 (35.1)	304 (48.2)	< 0.001
Mild-steatosis, n (%)	383 (12.25)	386 (8.69)	< 0.001	43 (7.1)	77 (11.9)	80 (13.0)	88 (14.0)	95 (15.1)	< 0.001
Severe-steatosis, n (%)	584 (18.68)	876 (19.72)	< 0.001	55 (9.1)	70 (10.8)	117 (19.0)	133 (21.1)	209 (33.1)	< 0.001

Q1, < 278 μmol/L; Q2, 278–317 μmol/L; Q3, 318–355 μmol/L; Q4, 356–402 μmol/L; Q5, ≥ 403 μmol/L

### Characteristics of men and women according to the quintiles of SUA levels

After stratification by the quintiles of SUA levels, the prevalence of hyper-ALT and the prevalence of NAFLD in men were elevated along with the increase of uric acid quintiles from 3.8%/16.3% at Q1 to 12.4%/48.2% at Q5 (*P*-values for trend< 0.001). Also, the prevalence of mild- and severe-steatosis was increased from 7.1/9.1% to 15.1/33.1% as the SUA levels elevated from Q1 towards Q5 (Table 1).

Similar characteristics were observed in women. Participants in the highest quintile of SUA (SUA ≥ 321 μmol/L) were older, with high BMI, SBP, and DBP. Serum biochemical parameters, such as TG, TC, LDL-C, AST, ALT, and FPG were positively associated with SUA levels, and HDL-C levels were negatively related to SUA levels (all *P*-values < 0.05).

Those participants with the highest quintile of SUA had high risks of hyper-ALT and NAFLD, which increased from 1.6 to 9.4% for hyper-ALT and from 9.4 to 49.6% for NAFLD as the SUA levels increased from Q1 to Q5 (all *P*-values< 0.001). Also, the prevalence of mild- and severe-steatosis elevated from 3.6 to 12.0% and 5.8 to 37.6%, respectively, along with the SUA levels elevated from Q1 to Q5 (Table 2).

**Table 2:** Characteristics of women according to the quintiles of SUA levels

Variables	Quintiles of SUA levels (mean ± SD)					*P*
	Q1 (n = 873)	Q2 (n = 881)	Q3 (n = 910)	Q4 (n = 878)	Q5 (n = 901)	
Age (years)	57.50 ± 9.23	58.63 ± 9.36	59.65 ± 9.17	61.23 ± 9.61	63.87 ± 10.41	< 0.001
BMI (kg/m^2^)	22.51 ± 2.73	23.37 ± 3.09	23.99 ± 2.88	24.69 ± 3.07	25.33 ± 3.44	< 0.001
SBP (mmHg)	136.01 ± 18.39	138.10 ± 19.70	141.96 ± 19.27	144.01 ± 19.41	148.44 ± 20.39	< 0.001
DBP (mmHg)	81.52 ± 11.77	82.53 ± 11.64	84.29 ± 10.56	85.73 ± 11.60	87.08 ± 11.85	< 0.001
TG (mmol/L)	1.06 ± 0.734	1.15 ± 0.63	1.29 ± 0.78	1.44 ± 0.76	1.68 ± 0.96	< 0.001
TC (mmol/L)	4.76 ± 0.87	4.85 ± 0.87	4.89 ± 0.89	5.02 ± 0.94	5.04 ± 0.92	< 0.001
HDL-C (mmol/L)	1.65 ± 0.39	1.59 ± 0.37	1.52 ± 0.37	1.45 ± 0.33	1.39 ± 0.34	< 0.001
LDL-C (mmol/L)	2.58 ± 0.66	2.69 ± 0.65	2.74 ± 0.69	2.86 ± 0.73	2.86 ± 0.71	< 0.001
AST (U/L)	21.01 ± 5.32	22.47 ± 9.81	22.79 ± 9.60	23.89 ± 9.28	25.22 ± 11.71	< 0.001
ALT (U/L)	16.57 ± 7.75	18.89 ± 16.35	19.71 ± 13.44	21.83 ± 13.53	22.86 ± 17.66	< 0.001
FPG (mmol/L)	5.96 ± 1.50	5.93 ± 1.16	5.91 ± 0.90	6.09 ± 1.26	6.15 ± 1.14	0.024
SUA (μmol/L)	185.58 ± 21.50	228.54 ± 8.94	260.37 ± 9.29	296.96 ± 12.55	378.07 ± 54.56	< 0.001
Hyper-ALT, n (%)	14 (1.6)	30 (3.4)	47 (5.2)	64 (7.3)	85 (9.4)	< 0.001
NAFLD, n (%)	82 (9.4)	152 (17.3)	226 (24.8)	355 (40.4)	447 (49.6)	< 0.001
M-steatosis, n (%)	31 (3.6)	59 (6.7)	82 (9.0)	106 (12.1)	108 (12.0)	< 0.001
S-steatosis, n (%)	51 (5.8)	93 (10.6)	144 (15.8)	249 (28.4)	339 (37.6)	< 0.001

Q1, < 213 μmol/L; Q2, 213–244 μmol/L; Q3, 245–276 μmol/L; Q4, 277–320 μmol/L; Q5, ≥ 321 μmol/L

*M-steatosis* mild-steatosis, *S-steatosis* severe-steatosis

### Associations of SUA levels with NAFLD

Results of multivariate logistic regression models showed a significant association between uric acid levels and elevated risk of NAFLD after adjustment for age and sex (*P *< 0.001). The ORs for the Q2 and Q5 compared to Q1 were 2.41 (95% CI 1.97–2.95) and 10.9 (95% CI 8.88–13.4), respectively. While the risk of NAFLD significantly increased by 5 times (OR = 5.26, 95% CI 4.01–6.90) for men and 10 times (OR = 10.2, 95% CI 7.80–13.3) for women as the SUA levels increased from Q1 to Q5. Further adjustment for BMI, history of cardiovascular related diseases, DBP, serum AST, TG, TC, HDL and FPG, the positive associations and linear trends between uric acid and prevalence of NAFLD were persisted (Table 3).

**Table 3:** Associations of SUA concentrations with NAFLD prevalence

SUA levels	Cases/n (%)	Model 1			Model 2		
		OR	95% CI	*P*	OR	95% CI	*P*
*Total (n* = *7569)*							
Q1	176/1523 (11.6)	1			1		
Q2	338/1536 (22.0)	2.41	1.97–2.95	< 0.001	1.78	1.41–2.23	< 0.001
Q3	473/1521 (31.1)	4.39	3.61–5.34	< 0.001	2.85	2.27–3.58	< 0.001
Q4	540/1481 (36.5)	6.31	5.16–7.72	< 0.001	3.19	2.52–4.04	< 0.001
Q5	702/15,084 (6.6)	10.92	8.88–13.44	< 0.001	4.34	3.39–5.54	< 0.001
*Men (n* = *3126)*							
Q1	98/603 (16.3)	1			1		
Q2	147/646 (22.8)	1.48	1.11–1.97	0.008	1.20	1.01–1.84	0.281
Q3	197/617 (31.9)	2.38	1.80–3.14	< 0.001	1.57	1.14–2.17	0.006
Q4	221/629 (35.1)	2.93	2.22–3.86	< 0.001	1.94	1.41–2.67	< 0.001
Q5	304/631 (48.2)	5.26	4.01–6.90	< 0.001	2.74	2.00–3.76	< 0.001
*Women (n* = *4443)*							
Q1	82/873 (9.4)	1			1		
Q2	152/881 (17.3)	2.04	1.53–2.71	< 0.001	1.70	1.24–2.34	0.001
Q3	226/910 (24.8)	3.27	2.48–4.29	< 0.001	2.27	1.67–3.08	< 0.001
Q4	355/878 (40.4)	6.83	5.23–8.91	< 0.001	4.07	3.02–5.48	< 0.001
Q5	447/901 (49.6)	10.19	7.80–13.31	< 0.001	4.60	3.39–6.24	< 0.001

Category of SUA quintiles

For all participants: Q1, < 231 μmol/L; Q2, 231–271 μmol/L; Q3, 272–312 μmol/L; Q4, 313–366 μmol/L; Q5, ≥ 367 μmol/L

For men: Q1, < 278 μmol/L; Q2, 278–317 μmol/L; Q3, 318–355 μmol/L; Q4, 356–402 μmol/L; Q5, ≥ 403 μmol/L

For women: Q1, < 213 μmol/L; Q2, 213–244 μmol/L; Q3, 245–276 μmol/L; Q4, 277–320 μmol/L; Q5, ≥ 321 μmol/L

Model 1: adjusted for age and sex (only for all participants)

Model 2: adjusted for age, sex (only for all participants), BMI, history of cardiovascular related diseases, DBP, serum AST, TG, TC, HDL and FPG

A positive association was observed between the SUA levels and the prevalence of mild- and/or severe-steatosis. Comparing to the first quintile, the ORs of mild steatosis increased from 1.70 (95% CI 1.24–2.32) to 3.48 (95% CI 2.50–4.86) as SUA levels increased from Q2 to Q5 for all participants, with the ORs of mild steatosis significantly increasing to 2.54 (95% CI 1.68–3.84) for men and 4.17 (95% CI 2.67–6.51) for women. Similar associations of SUA levels with severe-steatosis were observed for all participants, along with the increase of SUA levels to Q5, OR increased to 2.95 (95% CI 1.99–4.37) in men, and 4.93 (95% CI 3.41–7.12) in women, but the association was significant only for the Q3–Q5 in men, and the trend was persisted (Table 4).

**Table 4:** Associations of SUA concentration with the severity of Steatosis

SUA levels	Mild-steatosis				Severe-steatosis			
	Cases/n (%)	OR	95% CI	*P*	Cases/n (%)	OR	95% CI	*P*
Severity of steatosis (Model 1)								
*Total (n* = *7569)*								
Q1	72/1419 (5.1)	1			104/1451 (7.2)	1		
Q2	126/1324 (9.5)	2.10	1.55–2.84	< 0.001	212/1410 (15.0)	2.61	2.03–3.34	< 0.001
Q3	180/1228 (14.7)	3.67	2.74–4.92	< 0.001	293/1341 (21.8)	4.81	3.77–6.13	< 0.001
Q4	175/1116 (15.7)	4.16	3.07–5.64	< 0.001	365/1306 (27.9)	7.87	6.16–10.05	< 0.001
Q5	216/1022 (21.1)	6.52	4.78–8.88	< 0.001	486/1292 (37.6)	14.36	11.18–18.46	< 0.001
*Men (n* = *3126)*								
Q1	43/548 (7.8)	1			55/560 (9.8)	1		
Q2	77/576 (13.4)	1.76	1.19–2.62	0.005	70/569 (12.3)	1.25	0.86–1.83	0.239
Q3	80/500 (16.0)	2.20	1.48–3.26	< 0.001	117/537 (21.8)	2.51	1.78–3.56	< 0.001
Q4	88/496 (17.7)	2.66	1.80–3.92	< 0.001	133/541 (24.6)	3.14	2.23–4.43	< 0.001
Q5	95/422 (22.5)	3.75	2.54–5.53	< 0.001	209/536 (39.0)	6.44	4.62–8.98	< 0.001
*Women (n* = *4443)*								
Q1	31/822 (3.8)				51/842 (6.1)			
Q2	59/788 (7.5)	2.12	1.35–3.31	0.001	93/822 (11.3)	1.99	1.40–2.85	< 0.001
Q3	82/766 (10.7)	3.21	2.09–4.91	< 0.001	144/828 (17.4)	3.31	2.37–4.63	< 0.001
Q4	106/629 (16.9)	5.61	3.69–8.52	< 0.001	249/772 (32.3)	7.57	5.48–10.45	< 0.001
Q5	108/562 (19.2)	6.94	4.55–10.60	< 0.001	339/793 (42.7)	12.06	8.75–16.63	< 0.001
Severity of steatosis (Model 2)								
*Total (n* = *7569)*								
Q1	72/1419 (5.1)	1			104/1451 (7.2)	1		
Q2	126/1324 (9.5)	1.70	1.24–2.32	0.001	212/1410 (15.0)	1.86	1.39–2.48	< 0.001
Q3	180/1228 (14.7)	2.73	2.01–3.71	< 0.001	293/1341 (21.8)	2.98	2.24–3.96	< 0.001
Q4	175/1116 (15.7)	2.66	1.93–3.67	< 0.001	365/1306 (27.9)	3.71	2.77–4.96	< 0.001
Q5	216/1022 (21.1)	3.48	2.50–4.86	< 0.001	486/1292 (37.6)	5.18	3.83–7.01	< 0.001
*Men (n* = *3126)*								
Q1	43/548 (7.8)	1			55/560 (9.8)	1		
Q2	77/576 (13.4)	1.47	0.97–2.22	0.067	70/569 (12.3)	0.95	0.62–1.47	0.815
Q3	80/500 (16.0)	1.67	1.11–2.53	0.015	117/537 (21.8)	1.50	0.99–2.25	0.053
Q4	88/496 (17.7)	1.95	1.30–2.95	0.001	133/541 (24.6)	1.96	1.31–2.93	0.001
Q5	95/422 (22.5)	2.54	1.68–3.84	< 0.001	209/536 (39.0)	2.95	1.99–4.37	< 0.001
*Women (n* = *4443)*								
Q1	31/822 (3.8)	1			51/842 (6.1)	1		
Q2	59/788 (7.5)	1.89	1.20–2.98	0.006	93/822 (11.3)	1.58	1.06–2.35	0.025
Q3	82/766 (10.7)	2.42	1.55–3.75	< 0.001	144/828 (17.4)	2.22	1.52–3.24	< 0.001
Q4	106/629 (16.9)	4.02	2.61–6.20	< 0.001	249/772 (32.3)	4.21	2.92–6.05	< 0.001
Q5	108/562 (19.2)	4.17	2.67–6.51	< 0.001	339/793 (42.7)	4.93	3.41–7.12	< 0.001

Category of SUA quintiles

For all participants: Q1, < 231 μmol/L; Q2, 231–271 μmol/L; Q3, 272–312 μmol/L; Q4, 313–366 μmol/L; Q5, ≥ 367 μmol/L

For men: Q1, < 278 μmol/L; Q2, 278–317 μmol/L; Q3, 318–355 μmol/L; Q4, 356–402 μmol/L; Q5, ≥ 403 μmol/L

For women: Q1, < 213 μmol/L; Q2, 213–244 μmol/L; Q3, 245–276 μmol/L; Q4, 277–320 μmol/L; Q5, ≥ 321 μmol/L

Model 1: adjusted for age, sex (only for all participants)

Model 2: adjusted for age, sex (only for all participants), BMI, history of cardiovascular related diseases, DBP, AST, TG, TC, HDL and FPG

A significant linear increase manner for the prevalence of NAFLD with the elevation of ALT was observed in both men and women. The ALT-NAFLD association was stronger in men than in women, and the OR in comparing the Q4 to the Q1 was 9.51 for men (95% CI 7.42–12.19) and 7.99 for women (95% CI 6.49–9.84). All these trends and associations persisted after further adjustment for other confounders (Table 5).

**Table 5:** Associations of serum levels of ALT with the prevalence of NAFLD

ALT levels	Cases/n (%)	Model 1			Model 2		
		OR	95% CI	*P*	OR	95% CI	*P*
*Total (n* = *7569)*							
Q1	227/1899 (12.0)	1			1		
Q2	496/2259 (22.0)	2.09	1.76–2.48	< 0.001	1.54	1.26–1.87	< 0.001
Q3	482/1560 (30.9)	3.37	2.83–4.03	< 0.001	2.18	1.77–2.69	< 0.001
Q4	1024/1851 (55.3)	9.39	7.93–11.1	< 0.001	4.96	3.98–6.17	< 0.001
*Men (n* = *3126)*							
Q1	117/921 (12.7)	1			1		
Q2	153/684 (22.4)	1.93	1.48–2.52	< 0.001	1.38	1.02–1.86	0.04
Q3	264/805 (32.8)	3.16	2.47–4.04	< 0.001	2.03	1.53–2.68	< 0.001
Q4	433/716 (60.5)	9.51	7.42–12.2	< 0.001	4.76	3.47–6.53	< 0.001
*Women (n* = *4443)*							
Q1	160/1314 (12.2)	1			1		
Q2	188/924 (20.3)	1.85	1.47–2.32	< 0.001	1.44	1.11–1.88	0.006
Q3	401/1213 (33.1)	3.62	2.95–4.44	< 0.001	2.80	2.21–3.55	< 0.001
Q4	513/992 (51.7)	7.99	6.49–9.84	< 0.001	5.53	4.19–7.30	< 0.001

For all participants: Q1, < 14 U/L; Q2, 14–18 U/L; Q3, 19–24 U/L; Q4, ≥ 25 U/L

For men: Q1, < 16 U/L; Q2, 16–19 U/L; Q3, 20–27 U/L; Q4, ≥ 28 U/L

For women: Q1, < 14 U/L; Q2, 14–16 U/L; Q3, 17–23 U/L; Q4, ≥ 24 U/L

Model 1: adjusted for age and sex (just for all participants)

Model 2: adjusted for age, sex (only for all participants), BMI, history of cardiovascular related diseases, DBP, AST, TG, TC, HDL and FPG

### Joint associations of SUA and ALT with NAFLD prevalence

Subjects with both high ALT (> 40 U/L) and high SUA (> 420 μmol/L for man, > 360 μmol/L for woman) levels had a 27-time higher prevalence of NAFLD than subjects with both low ALT and low SUA levels after adjustment for age and sex among all participants, further adjustment of other confounders did not change the association (Table 6). Associations of both high ALT and high SUA with NAFLD was significant in both men and women, but the joint associations seemed to be slightly higher in men than in women, with the OR in full-models 10.8 (95% CI 3.56–32.5) for men and 7.96 (95% CI 2.83–22.4) for women.

**Table 6:** Joint associations of serum ALT and SUA with NAFLD prevalence

Group	Cases/n (%)	Model 1			Model 2		
		OR	95% CI	*P*	OR	95% CI	*P*
*Total (n* = *7569)*							
Low ALT + low SUA	1500/6269 (82.8)	1					
Low ALT + elevated SUA	411/840 (11.1)	3.22	2.77–3.74	< 0.001	1.94	1.61–2.34	< 0.001
Elevated ALT + low SUA	232/364 (4.8)	5.34	4.27–6.67	< 0.001	2.99	2.17–4.11	< 0.001
Elevated ALT + elevated SUA	86/96 (1.3)	26.75	13.85–51.65	< 0.001	9.15	4.31–19.40	< 0.001
*Men (n* = *3126)*							
Low ALT + low SUA	613/2482 (79.4)	1					
Low ALT + elevated SUA	187/408 (13.1)	2.90	2.33–3.61	< 0.001	1.99	1.53–2.59	< 0.001
Elevated ALT + low SUA	119/184 (5.9)	4.87	3.54–6.71	< 0.001	2.78	1.81–4.25	< 0.001
Elevated ALT + elevated SUA	48/52 (1.7)	30.68	10.97–85.77	< 0.001	10.75	3.56–32.46	< 0.001
*Women (n* = *4443)*							
Low ALT + low SUA	887/3787 (85.2)	1					
Low ALT + elevated SUA	224/432 (9.7)	3.48	2.83–4.28	< 0.001	2.00	1.55–2.58	< 0.001
Elevated ALT + low SUA	113/180 (4.1)	5.55	4.06–7.58	< 0.001	3.18	1.99–5.08	< 0.001
Elevated ALT + elevated SUA	38/44 (1.0)	20.60	8.68–48.90	< 0.001	7.96	2.83–22.39	< 0.001

Elevated ALT: ALT > 40 U/L; Elevated SUA: Men: > 420 μmmol/L, Women: > 360 μmmol/L

Low ALT: ALT ≤ 40 U/L; Low SUA: Men: ≤ 420 μmmol/L, Women: ≤ 360 μmmol/L

Model 1: adjusted for age and sex (only for all participants)

Model 2: adjusted for age, sex (only for all participants), BMI, history of cardiovascular related diseases, DBP, AST, TG, TC, HDL and FPG

## Discussion

Our study found that SUA was positively associated with NAFLD in both men and women, whereas the association was slightly stronger in women. Furthermore, significant joint associations of SUA and ALT with NAFLD prevalence were observed in all participants, and subjects with low ALT and high SUA had a significantly higher prevalence of NAFLD than those with both low ALT and low SUA levels, which suggested that SUA might be an independent risk factor for NAFLD. Meanwhile, the joint associations were slightly stronger in men than in women.

The positive associations between SUA levels and NAFLD in our study were in concordance with previous epidemiological studies in Asia [25, 26]. Results were observed in non-diabetic Chinese men [27] and in healthy Japanese population [28]. A meta-analysis also supports the positive association of hyperuricemia with risk of NAFLD in Asian populations [29]. Similar results from the National Health and Nutrition Examination Survey 1988–1994 in the United States also found a positive association between uric acid levels and NAFLD in non-diabetic adults [30].

According to Younossi’s study [31], the NAFLD prevalence in Asia ranges from 15 to 40%, and the reported prevalence of fatty liver has also increased in many regions of China increased from 3.87% in 1995 to 14.04% in 2002, 17.3% in 2005, and 43.65% in 2015 among Shanghai adults. Meanwhile, school children also became the population at higher risk of NAFLD now, 5.0% of 7229 school children from the Yangtze River delta region in China had NAFLD (7.5% in boys, 2.5% in girls) [32]. Thus, it is imperative to explore the exact cause of NAFLD.

Evidence from epidemiological studies indicated a sex difference for NAFLD prevalence. Women have a relative lower prevalence rate in adulthood compared to men, then the prevalence rises after the age of 50 years, reaches to the peak at 60–69 years, and declines after 70 years [19], while the prevalence of NAFLD showed an inverted U-shape for men, which increased in adulthood, then declined significantly after age of 50–60 years [19, 20]. Literatures show that, postmenopausal women have a similar [33, 34] or even higher prevalence of NAFLD compared to men at the same age owing to ovarian senescence and estrogen deficiency [35, 36], thus, the higher prevalence of NAFLD in those elderly women included in our study might be the combined effects of decreased estrogen levels and increased SUA levels.

ALT is a well-established marker of liver inflammation and hepatocellular injury, and usually used to predict the development and regression of fatty liver. One cross-sectional study based on a larger sample (including 82,608 adults) suggested the stronger association of SUA with elevated ALT in women than in men, and the similar associations was found among NAFLD patients as the SUA level was higher than 5.6 mg/dL [21]. In the present study, SUA showed a stronger association with NAFLD prevalence in women, and the association between ALT and NAFLD prevalence was stronger in men. Meanwhile, the elevated levels of SUA combined with ALT corresponded to the prevalence of NAFLD for both males and females, but the associations were slightly stronger in men, suggesting that subjects with both elevated levels of SUA and ALT should be a target population for the prevention of NAFLD.

Although there is evidence suggesting the involvement of uric acid in NAFLD’s pathogenesis, the question that remains is whether hyperuricemia is a causal factor in NAFLD or merely a marker for its presence. Evidence also supported uric acid as a powerful antioxidant [37], which could scavenge peroxynitrite and peroxynitrite-derived radicals [38]. Therefore, the increased levels of SUA might be the compensatory reaction of SUA to counteract the oxidative stress, but not the cause of NAFLD. NAFLD patients with elevated ALT might already have much more serious damage of liver. Thus, the elevated levels of ALT would induce a higher risk of NAFLD than elevated levels of SUA.

This is the first study to investigate the sex-specific joint associations of SUA and ALT with NAFLD prevalence in elderly Chinese. However, the present study has some limitations. First, the diagnosis of NAFLD was conducted by ultrasonographic examination instead of liver biopsy, which might omit mild steatosis. However, ultrasonographic examination is widely used to screen NAFLD in epidemiological research as it is non-invasive, safe, widely available and portable, and the sensitivity and specificity for detecting hepatic steatosis is acceptable; Second, the assessment of NAFLD degree was conducted by ultrasound, which might induce subjectivity and inconsistency. Although ultrasound might not be the golden standard evaluator for NAFLD degree, liver ultrasonography is a noninvasive method which is most commonly used to evaluate the degree of steatosis for NAFLD in the practice [39]. To reduce the subjectivity and improve the consistency between the assessments of ultrasound physicians, all experienced physicians involved in our study were well-trained. Third, our present study had excluded patients who had self-reported taking anti-hypertensive, anti-diabetic, lipid lowering or hypouricemic agents on the day of medical examination, those patients were at higher risk of NAFLD; Fourth, the cross-sectional nature of our study cannot test the causal relationship between SUA, ALT and NAFLD risk, further well-designed and large-scaled prospective studies are required to speculate on our conclusions.

## Conclusions

In our study, we found that there was a significant positive association between SUA and NAFLD in both men and women, and the association was slightly stronger in women. Furthermore, significant joint associations of SUA and ALT with NAFLD prevalence were observed in all participants. Our findings suggested the combination of serum SUA and ALT might have potential clinical values for the earlier prevention and screening of NAFLD. Further well-designed and large-scaled prospective studies are warranted to confirm these findings.

## Abbreviations

ALT: alanine aminotransferase
AST: aspartate aminotransferase
BMI: body mass index
BP: blood pressure
DBP: diastolic blood pressure
FPG: fasting plasma glucose
HDL-C: high-density lipoprotein cholesterol
LDL-C: low-density lipoprotein cholesterol
NAFLD: non-alcoholic fatty liver disease
ORs: odds ratios
SUA: serum uric acid
